# Self-assembling peptides as immunomodulatory biomaterials

**DOI:** 10.3389/fbioe.2023.1139782

**Published:** 2023-03-01

**Authors:** Andrea Hernandez, Jeffrey D. Hartgerink, Simon Young

**Affiliations:** ^1^ Katz Department of Oral and Maxillofacial Surgery, The University of Texas Health Science Center at Houston, School of Dentistry, Houston, TX, United States; ^2^ Department of Chemistry and Department of Bioengineering, Rice University, Houston, TX, United States

**Keywords:** biomaterials, self-assembling peptides (SAP), cancer, immunotherapy, localized delivery

## Abstract

Self-assembling peptides are a type of biomaterial rapidly emerging in the fields of biomedicine and material sciences due to their promise in biocompatibility and effectiveness at controlled release. These self-assembling peptides can form diverse nanostructures in response to molecular interactions, making them versatile materials. Once assembled, the peptides can mimic biological functions and provide a combinatorial delivery of therapeutics such as cytokines and drugs. These self-assembling peptides are showing success in biomedical settings yet face unique challenges that must be addressed to be widely applied in the clinic. Herein, we describe self-assembling peptides’ characteristics and current applications in immunomodulatory therapeutics.

## 1 Introduction

An influx of attention has been drawn to self-assembling peptides as the organization of these nanostructures has made it possible to interact with biological systems and induce bioactivity necessary for applications in tissue engineering, cancer immunotherapy, drug delivery, and vaccine design. Mainly, in tissue engineering, it was common to use scaffolds derived from animal or plant sources such as collagen, chitosan, and alginate (Loo et al., 2015; Peng et al., 2022). These scaffolds can potentially induce immunogenicity (Berthiaume et al., 2011; Loo et al., 2015; Andorko and Jewell, 2017). Self-assembling peptides (SAPs) can mimic tissues’ natural biomechanics and structure and the extracellular matrix (ECM). Besides the self-assembling peptides’ natural framework, SAPs are tunable to include biologically active systems. The adaptable nature of these peptides allows for loading a wide range of modulators that can be modified through their chemical composition to stabilize the scaffold, control release, and influence biological activity (Hoffman, 2002; Habibi et al., 2016; Sankar et al., 2021). SAPs are attractive biomaterials that can deliver immunomodulators known to have rapid clearance and poor metabolic stability (Lee et al., 2019; La Manna et al., 2021). The delivery site of these modulators can range from intracellular to distant targeted tissues. SAPs’ potential in tissue engineering, drug delivery, and immunotherapy can change the way we design and deliver biologics.

**FIGURE 1:**
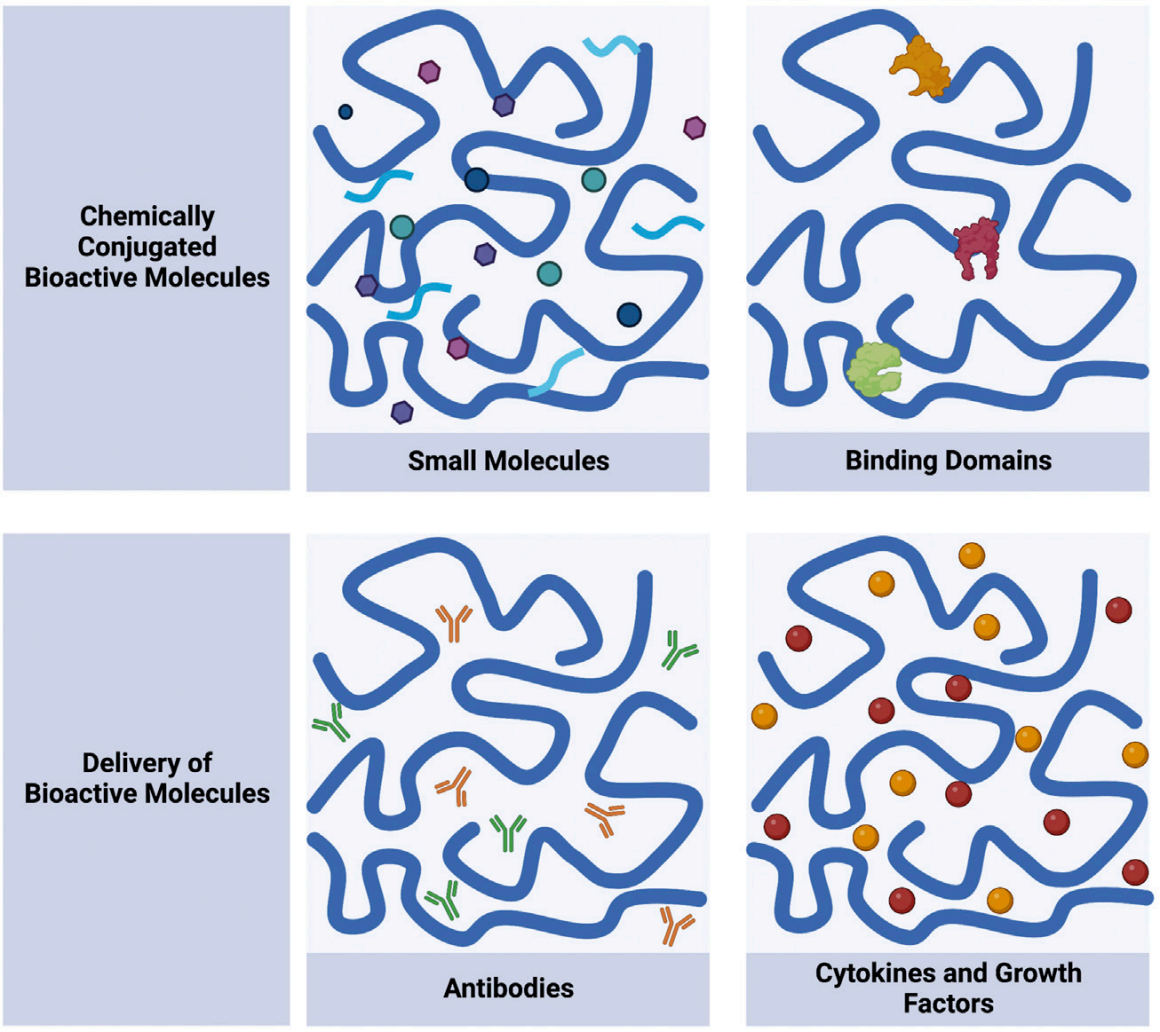
Self-assembling peptides can be designed to chemically conjugate and deliver bioactive molecules. Chemically conjugated molecules include small molecules and binding domains. Delivery of molecules includes antibodies and cytokines and growth factors. Schematic was made using Biorender.com.

Many SAPs form networks of nanofibers that generate hydrogels at low weight percent. Some of the SAP hydrogels have shear-thinning and self-healing properties, which broadens the scope of using them as a biomaterial. Shear-thinning is a material’s ability to decrease viscosity under shear strain and self-heal when the strain is removed (Diesendruck et al., 2015; Chen et al., 2018a). Hydrogels with shear-thinning and self-healing properties are attractive biomaterials because they can be injected for minimally-invasive delivery (Herbst et al., 2013; Chen and Zou, 2019). Upon injection, the hydrogel can recover to the shape of the local environment, an important property for scaffolds in tissue engineering (Overstreet et al., 2012; Uman et al., 2020). These hydrogels experience improved material retention and mechanical properties that allow homogenous sequestration of modulators, making them ideal for the controlled release of small molecules for drug delivery (Lopez-Silva et al., 2019; Chang and Yan, 2020; Han et al., 2020; Uman et al., 2020). Maintaining biomaterial integrity is vital to restore vasculature and bone (Davison et al., 2014; Williams, 2019). Over time, the material can erode, but the early roots of regeneration are necessary to build the foundation of tissue growth (Berthiaume et al., 2011; O'Brien, 2011). Similarly, controlled material degradation is also required to ensure the release of immunomodulators for drug delivery and immunotherapy applications (Langer et al., 1990; Zhang et al., 2018). This review will cover self-assembling peptides’ unique contribution to biological activity and as delivery vehicles (Figure 1). SAPs will be discussed as small molecule mimickers and binding domains that contribute to host response and as delivery vehicles for immunomodulators such as antibodies and cytokines. The mechanism of peptide assembly and their chemical modifications have been recently reviewed in great detail and will not be discussed here (Lee et al., 2019; La Manna et al., 2021; Sinha et al., 2021; Li et al., 2022).

## 2 Designing self-assembling peptides for immunomodulation

Self-assembling peptides are a class of peptides in which numerous copies interact with one another through a variety of non-covalent interactions to generate specific nano- and micro-structures in a fashion reminiscent of Lego Blocks. Numerous SAP “building blocks” have been described, each with its own unique characteristics and design criteria for self-assembly into nanostructures which have been reviewed in-depth (Lee et al., 2019; La Manna et al., 2021). A large number of SAPs are being explored for their potential in biomedical applications. In particular, MultiDomain Peptides (MDPs), Self-Assembling Amphiphilic Peptide Drug Conjugate (SAAPDC), RADARADARADARADA (RADA16), KLDLKLDLKLDL (KLD-12), SSGGPLGVRGKLVFFCAWSATWSNYWRH (LMY1), QAR-ILEADAEILRAYARILEAHAEILRAD (Coil-29), AEAEAKAKAEAEAKAK (EAK16-II), and QQKFQFQFEQQ (Q11) with their respective current immunotherapy strategies will be reviewed (Table 1) (Tripathi et al., 2015; Ding et al., 2016; Moore and Hartgerink, 2017; Mora-Solano et al., 2017; Ji et al., 2018; Wang et al., 2019; Lv et al., 2022; Wu et al., 2022).

**TABLE 1:** Overview of reviewed self-assembling peptide immunotherapy strategies.

Material type	Immunotherapy strategy	Results and application	Reference
RADA16	PD-1, DCs and tumor antigens in RADA16	Supported DC efficacy and antitumor activity	Yang et al. (2018)
KLD12	TNF-α neutralizing antibody and HGF in KLD2R/heparin hydrogel	Enhanced renal protective potential and reduced chronic renal fibrosis	Liu et al. (2020)
MDP	L-NIL drug mimic in MDP	Reduced nitrotyrosine levels in and around the implant	Leach et al. (2019a)
	CDN sequestered in L-NIL-MDP	Decreased tumor growth and prolonged survival	Leach et al. (2021)
	CCL2-binding moiety to form SLaM	Reduced monocyte migration, and sequestered CCL2 molecules	Kim et al. (2020)
	MCP-1 and IL-4 in SLac	Promoted infiltration and polarization of macrophages to M2 phenotype	Kumar et al. (2015)
SAAPDC	DOX-KGFRWR in SAAPDC	Prolonged survival and antitumor response	Ji et al. (2018)
LMY1	CD47 targeting motif, MMP-2 motif and PD-1 in LMY1	Improved survival outcomes and antitumor response	Lv et al. (2022)
Coil-29	PEPvIII, TRP2, and Toxoid	Prolonged survival and inhibited tumor growth	Wu et al. (2022)
EAK16-II	SL9 to form SL9-EAK16-II and TLR 7/8 agonist	Developed strong SL9 specific CD8 T cell responses	Ding et al. (2016)
Q11	TNFQ11/PADREQ11 and TNFQ11/VACQ11	Improved survival in TNF-mediated inflammation model	Mora-Solano et al. (2017)

As an example, MDPs are a class of SAP that form nanofibers consisting of a bilayer of β-sheet hydrogen bonded peptides. This organization is facilitated due to the amphiphilic nature of the MDP with hydrophilic amino acids facing the aqueous solution and hydrophobic amino acids facing the interior of the fiber bilayer. In this arrangement, the peptides form hydrogen bonds down the length of the fiber to further stabilize the assembly. Charged residues at the peptide’s termini are frequently designed to be negatively or positively charged, and this charge can be used to control assembly.

Recently four different charged MDPs were compared *in vivo* for their reaction with the host immune system (Moore et al., 2018; Lopez-Silva et al., 2020). In a murine subcutaneous injection model, the positively charged lysine-based MDP, K_2_(SL)_6_K_2_ (K_2_ MDP), and arginine-based MDP, R_2_(SL)_6_R_2_ (R_2_ MDP) promoted an inflammatory response compared to the negatively-charged E_2_(SL)_6_E_2_ (E_2_ MDP) and D_2_(SL)_6_D_2_ (D_2_ MDP). Through histological analysis, both amine groups K_2_ MDP and R_2_ MDP saw infiltration by host cells. K_2_ MDP was homogenously infiltrated by host cells, while R_2_ MDP had heterogeneous infiltration with a higher total number of cells and distinct cell pools. The SAPs with carboxylate side chains, E_2_ MDP and D_2_ MDP, had less infiltration. From early time points in flow cytometry and tSNE evaluations, K_2_ MDP had the presence of monocytes and macrophages, which resolved over time, indicating an acute inflammatory response. Meanwhile, R_2_ MDP had more polymorphonuclear myeloid-derived suppressor cells and, over time, did not resolve, implying chronic inflammatory reactions. These results provide a greater understanding of developing SAPs for particular purposes. In the case of tissue regeneration, K_2_ MDP would be a better SAP option as, over time, the material degrades, and inflammation declines. R_2_ MDP would be advantageous in cancer immunotherapy because having localized inflammation in the tumor immune microenvironment stimulates antitumor responses from macrophages that produce inflammatory cytokines like interleukin-1β (IL-1β), IL-6, and tumor necrosis factor-α (TNF-α) and lymphocytes such as natural killer cells, T cells, and B cells that induce cytotoxic activity (Chen et al., 2018b). E_2_ MDP and D_2_ MDP can be helpful in cases where the material does not provoke an immune response towards transplanted therapies such as mouse pancreatic-islet-derived cells for the treatment of diabetes (Su et al., 2010; Griffith et al., 2016). Thus, when developing SAPs, a significant consideration should be the hydrogel’s amino acid sequence to achieve the desired immune response.

SAPs can be chemically conjugated with small molecules and biologic recognition motifs to enhance cellular uptake (Hoffman, 2002; Wu et al., 2012; Fan et al., 2017; Levin et al., 2020). The alteration of the peptide primary sequence can include short bioactive amino acid sequences capable of initiating a biological response in the termini or, in some cases, in the middle of the sequence of SAPs. Cells can adhere to the nanostructures since most SAPs resemble the ECM. Cell adhesion-promoting bioactive amino acid sequences such as RGD can stimulate cell adherence (Cui et al., 2010). SAPs should be customized to the application and responsive to the environment. For instance, in bone defect repair, the rate of material degradation to the rate of bone regeneration is essential for developing new bone tissue (Dumas et al., 2014; Wei et al., 2020). SAPs can be remodeled by integrating sites in the material which are cleavable by enzymes such as matrix metalloproteinases**-**2 (MMP-2). These cleavable sites will allow healthy tissue to break down the material to make way for new tissue formation. In cancer applications, to prevent degradation of the ECM and metastasis, MMP-2 can be inhibited (Ji et al., 2018). SAPs can bind to cell surface receptors to activate or inhibit their signal. SAPs can include angiogenic cell surface receptor agonists to promote tissue development, such as VEGF mimic sequence (Siddiqui et al., 2021). Additionally, anti-angiogenic domains can prevent neovascularization (Nguyen et al., 2018). The signal is contained and localized in the gel by incorporating small molecules and biologic recognition domains in the hydrogel.

SAPs can load bioactive molecules for local delivery. During hydrogel formation, the SAPs can include the substances of interest. For instance, molecules such as drugs and cytokines can diffuse out of the gel. The rate at which the molecule diffuses out depends on the molecule’s identity and is not yet fully understood (Moore and Hartgerink, 2017). SAPs can load different polar molecules. For example, hydrophobic molecules are sequestered in the hydrophobic core of the SAP, while hydrophilic molecules are in the hydrophilic regions of the SAP. The ability to modify peptides and allow for the natural assembling process makes SAPs appealing for nanomaterial production.

## 3 Self-assembling peptide mimicking biological functions

Self-assembling peptides’ nanostructures resemble the ECM and can modify their peptide sequence for broad applications. Bioactive amino acid sequences, cleavable sites, and receptor agonists in SAPs can modulate the immune system (Hoffman, 2002; Wu et al., 2012; Fan et al., 2017). SAPs allow the signal to be contained and localized in the gel by incorporating small molecules and biologic recognition domains.

### 3.1 Small molecules

Engineered self-assembling peptides can deliver small molecules to targeted sites. Typically, small molecules are low in molecular weight and capable of modulating biochemical processes for diagnosis, treatment, or disease prevention (Lu and Atala, 2014). Most small molecules can cross the cell membrane allowing them to target intracellular proteins, and can be produced in large quantities, making them appealing for large-scale manufacture. However, most small molecules do not have targeting capability making it difficult to avoid off-target effects and maintain stability (Kidane and Bhatt, 2005; Gurevich and Gurevich, 2014). Researchers overcome this barrier by integrating small molecule mimics as a functional group in SAPs.

**FIGURE 2:**
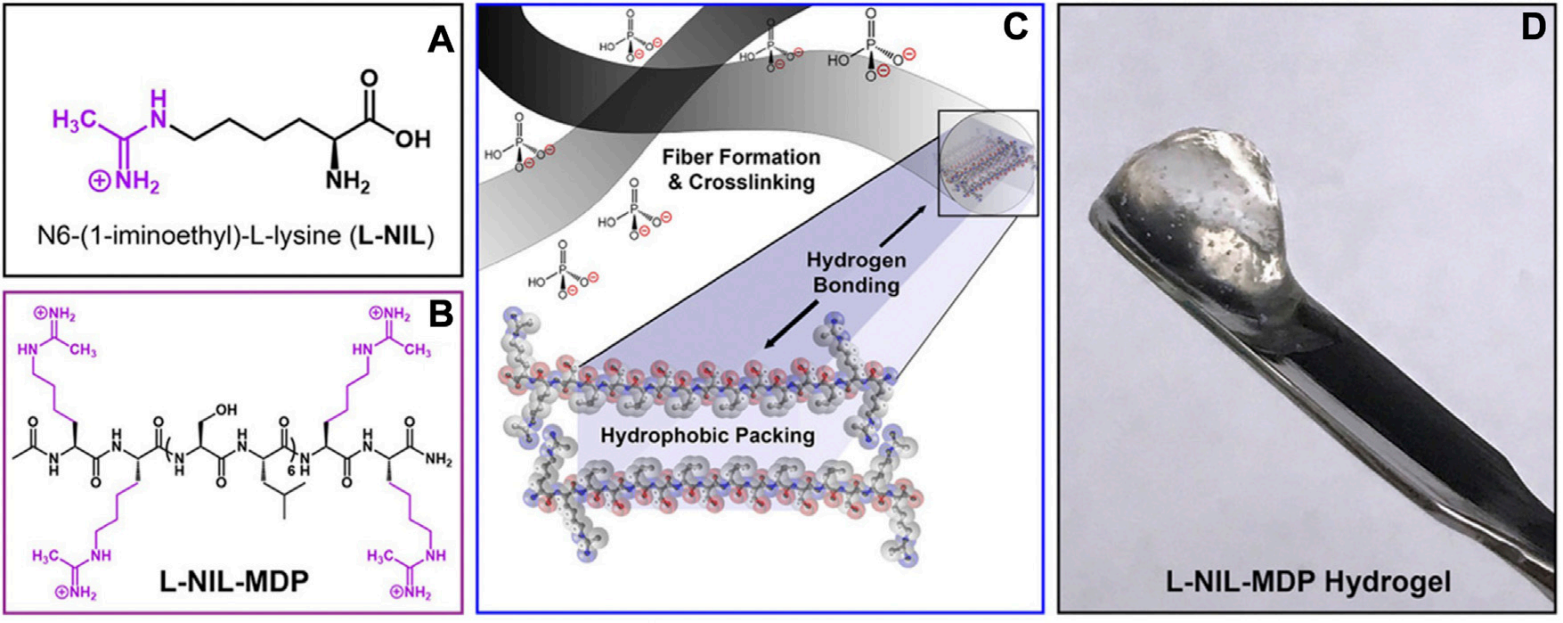
Self-assembling peptide chemical structure of N^6^-(1-iminoethyl)-L-lysine **(A)** Design of L-NIL-MDP. **(B)** Lysine side chains from starting material, K_2_(SL)_6_K_2_, converted to L-NIL functional group. **(C)** Schematic demonstrating the nanofibers formed from MDPs into antiparallel β-sheets. **(D)** Image of L-NIL-MDP formed at 1 wt% in a phosphate-containing buffer. Reprinted with permission from Leach et al. (2019b). Copyright 2019 American Chemical Society.

When designing biomaterials for biomedical applications, it is critical to tailor them to the disease. In the case of cancer, for example, there is an upregulation of inducible nitric oxide synthase (iNOS) in the tumor immune microenvironment (Jenkins et al., 1995; Zhang and Xu, 2001). Some researchers have used a small molecule inhibitor, N^6^-(1-iminoethyl)-L-lysine (L-NIL), to target iNOS to achieve tumor regression (Jenkins et al., 1995; Zhang and Xu, 2001; Fukumura et al., 2006; Jayaraman et al., 2012). An MDP with L-NIL-like functionality, K^LNIL^_2_(SL)_6_K^LNIL^_2_ (L-NIL-MDP), was developed to mimic a small-molecule inhibitor with immunomodulatory properties (Figure 2). L-NIL-MDP *in vitro* inhibited iNOS activity and nitrite production in cell populations interacting with the hydrogel material (Leach et al., 2019a). From a single subcutaneous injection of L-NIL-MDP, immunohistochemistry showed L-NIL maintained low nitrotyrosine levels in and around the implant over 7 days.

Additionally, the modified L-NIL-MDP with inherent bioactivity can be synergistically combined with a controlled released small molecule, cyclic dinucleotide (CDN). CDNs can induce antitumor responses in preclinical models through the Stimulator of Interferon Genes (STING) pathway (Corrales et al., 2015; Leach et al., 2018). The STING pathway links the detection of cytosolic tumor DNA through the enzyme cyclic GMP-AMP synthase, which activates STING resulting in the upregulation of type I interferons (Leach et al., 2019b; Jiang et al., 2020; Decout et al., 2021). This signaling allows for the crosstalk between tumor and immune cells, which promotes antitumor responses (Jiang et al., 2020). Clinical trials have evaluated intratumoral injections of CDN as a monotherapy, such as ADU-S1007 and MK14548 (ClinicalTrials.gov: NCT03172936, NCT03010176) and a modified CDN that forms a macrocycle-bridged STING agonist (Kim et al., 2021). These efforts may not be sufficient to localize the delivery of these small molecules. L-NIL-MDP was used to sequester CDN for controlled administration (Leach et al., 2021). Tumor-bearing mice treated with the L-NIL-MDP + CDN group showed decreased tumor growth compared with K_2_ MDP + CDN treatment. Survival increased with the L-NIL-MDP + CDN group compared to a saline control, L-NIL-MDP alone, and K_2_ MDP alone. Currently, the mechanism of action for L-NIL-MDP is being investigated.

**FIGURE 3:**
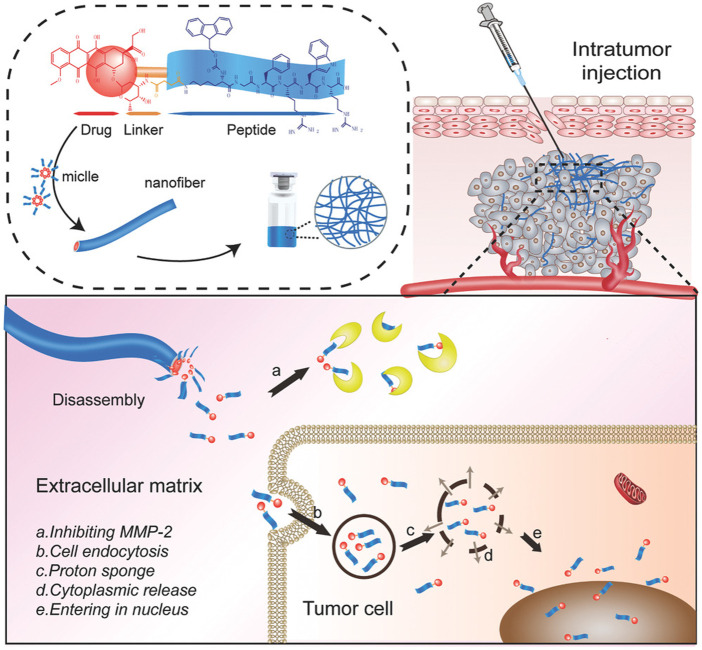
Self-assembling amphiphilic peptide drug containing amphiphilic peptide drug conjugate from oligomeric peptides (KGFRWR) and MMP-2 inhibitor doxorubicin (DOX). Reprinted from Ji et al. (2018).

Other SAPs have also been used for promoting antitumor activity. An SAAPDC was created containing amphiphilic peptide drug conjugate from oligomeric peptides (KGFRWR) and MMP-2 inhibitor doxorubicin (DOX) to treat hepatocellular carcinoma (Figure 3). DOX-KGFRWR strengthened the inhibition of MMP-2 activity and sustained the release of DOX in the DOX-KGFRWR group compared to DOX alone (Ji et al., 2018). The *in vivo* antitumor efficacy studies showed prolonged survival and therapeutic efficacy in the DOX-KGFRWR group. DOX-KGFRWR was able to control the release and retention of DOX without inducing severe systemic toxicity. The hexapeptide-based supramolecular system illustrated local and controlled drug release profiles. Researchers should explore the combination of embedding functional motifs and sequestering small molecules into SAPs in cases where synergy is required to improve response.

### 3.2 Binding domains

Besides integrating small molecules, SAPs can incorporate binding domains to activate or inhibit cellular processes. Parallel and serial binding are two types of interactions common in receptor binding. In the case of parallel binding, coinciding binding occurs between multiple sites on the molecule and can have stronger total binding strength than monovalent interactions (Mammen et al., 1998; Varner et al., 2015). On the other hand, serial binding involves repeated weak-binding events that result in biological activities such as signal transduction (Ohlson et al., 1997; Ohlson, 2008). These binding activities play a fundamental role in intracellular and extracellular activity. Therefore, researchers can incorporate the binding domains in SAPs to get a biologic response. There are non-self-assembling therapeutic peptides that can deliver immunomodulatory agents. Such therapeutic peptides have been thoroughly reviewed and will not be discussed here (Lau and Dunn, 2018; Li et al., 2021; Thakur et al., 2022).

More than one binding domain can be incorporated into SAPs for vaccine applications. Particularly in active immunotherapy, the patient’s immune system is stimulated to produce a therapeutic response toward a disease or pathogen. The main goal of active immunotherapy is to generate a predictable B cell response without an autoreactive T cell response (Jia et al., 2013). Researchers have used B cell epitopes from the targeted protein, non-autologous T-helper epitopes incorporated in a carrier protein, and if the response is insufficient, adjuvants are added (Durez et al., 2014; Bavoso et al., 2015; Zhang et al., 2016). A supramolecular peptide system was created with exogenous T-cell epitopes and TNF B cell epitopes co-assembled into a nanofiber without additional adjuvants (Mora-Solano et al., 2017). Two T-cell epitopes were generated: high-affinity universal CD4^+^ T-cell epitope, PADRE, was used with Q11 to form PADREQ11, and T-cell epitope from Vaccinia I1L protein was used with Q11 to create VACQ11. A B cell epitope from mouse TNF was used with Q11 to form TNFQ11. In a TNF-mediated inflammation model, lipopolysaccharide was delivered intraperitoneally, and mice immunized with TNFQ11/PADREQ11 or TNFQ11/VACQ11 had improved survival. These findings indicate that a combination of B and T cell epitopes can produce immune cells’ specific activation towards a disease state.

Binding domains and small molecules can be included in SAPs. For instance, EAK16-II incorporated HIV-1 specific cytotoxic T-lymphocyte (CTL) epitope, SL9, to form SL9-EAK16-II (Ding et al., 2016). Additionally, toll-like receptor (TLR) agonists, R848 or R837, were included in SL9-EAK16-II as they have been shown to reduce the ability of lymphoid tissue to support HIV infection (Hofmann et al., 2016; Bam et al., 2017). *In vitro* and *in vivo* studies showed the co-delivery of CTL epitope and TLR7/8 agonist group SLP-EAK16-II/R848 had significantly strong SL9-specific CD8 T cell responses. A combination of known epitopes and agonists can be delivered through SAPs to obtain a desired immune response.

## 4 Self-assembling peptides releasing immunomodulators

Several new immunomodulators, such as immune checkpoint inhibitors (ICIs) and cytokines, have been used in a wide array of research areas. Immunomodulators can be divided into immunostimulators and immunosuppressants; depending on the context, they can activate or prevent immune cell activity. These modulators may face insufficient immune stimulation, off-target side effects, and bioactivity loss during circulation (Sathish et al., 2013; Feng et al., 2019). Many of these modulators, whether monotherapeutic or in combination, are administered systemically and require high doses to maintain modulators in circulation, ultimately resulting in high toxicities (Bast et al., 2019; Wang et al., 2022). Therefore, it is vital to have localized and sustained release of these immunomodulators. Many researchers have begun using self-assembling peptides to release immunomodulators, which have shown promise in several fields.

### 4.1 Antibodies

ICIs have succeeded in melanoma, renal cell carcinoma, and non-small cell lung cancer (Das and Johnson, 2019; Franzin et al., 2020). In head and neck squamous cell carcinoma (HNSCC), ICIs have shown some success. Particularly, anti-programmed death 1 (PD-1) was approved for treating recurrent/metastatic HNSCC (Burtness et al., 2019). Even though HNSCC has a similar mutational and immune profile as other solid cancers, anti-cytotoxic T lymphocyte-associated antigen 4 (CTLA-4) failed to demonstrate a benefit for HNSCC patients. Most of the HNSCC clinical trials testing anti-CTLA-4 recruited patients who previously received ablative locoregional therapies since HNSCC is prone to regional lymphatic metastasis (D'Cruz et al., 2015; Chow, 2020; Mody et al., 2021). Recently, it was found that by preserving the lymphatics, there is a robust immune response with anti-CTLA-4 in an HNSCC murine model (Saddawi-Konefka et al., 2022). Using a combination of anti-CTLA-4 and immunomodulators known to drive antigen processing and cross-presentation can help mount a robust antitumor response.

Tumor characteristics such as tumor immune infiltration and DNA damage pathways can influence immune checkpoint inhibitor efficacy (Bai et al., 2020; Meyers and Banerji, 2020). Tumor cells’ mutational burden can increase tumor antigenicity and enhance evasion strategies to targeted treatments (Seidel et al., 2018). Therefore, immunostimulatory substances are crucial for ICI combinations to have antigenicity and adjuvanticity (Galluzzi et al., 2020; Kwon et al., 2021). For instance, there have been early reports of combining anti-CTLA-4 with immunostimulatory substances such as GM-CSF, anti-CD25, and anti-CD40 (van Elsas et al., 1999; Sutmuller et al., 2001; Quezada et al., 2006; Sorensen et al., 2010). In an Oral Squamous Cell Carcinoma mouse model, anti-PD-1 with a STING agonist had improved tumor response compared to PBS + IgG2a (Shi et al., 2022). With these combinations, it was common to administer ICIs with their respective immunostimulant frequently. A biomaterial like SAPs can provide a controlled release strategy for ICIs and incorporate other motifs for targeting.

**FIGURE 4:**
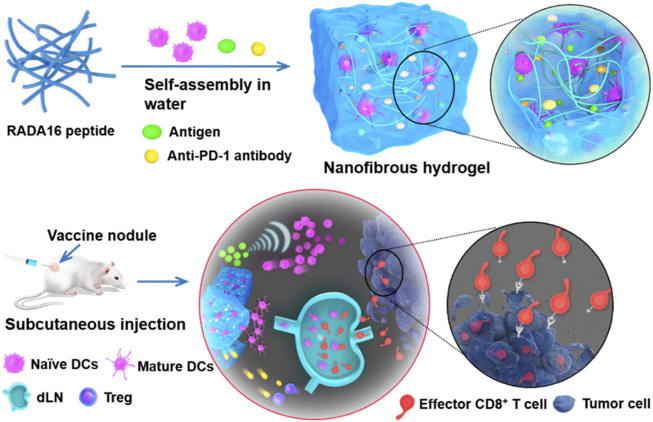
Delivery of exogenous dendritic cells and PD-1 in RADA16 for immunotherapy vaccine. Reprinted with permission from Yang et al. (2018). Copyright 2018 American Chemical Society.

SAPs can load ICIs and other substances that elicit an antitumor response. As an illustration, a mixture of RADA16 peptide, anti-PD-1 antibodies, DCs, and tumor antigens was developed for cancer treatment (Figure 4). RADA16 peptide DC with model tumor antigen ovalbumin (OVA) and anti-PD-1 antibody (Gel-DC-OVA + anti-PD-1) increased the proportion of activated DCs in the draining lymph nodes and reduced regulatory T cells (T-regs) (Yang et al., 2018). Mice were rechallenged with, EG7-OVA cells, which are mouse lymphoma cells that exogenously expressed ovalbumin, and they found that Gel-DC-OVA and Gel-DC-OVA + anti-PD-1 delayed tumor growth. Although there were no significant differences in the addition of PD-1 blockade compared to Gel-DC-OVA, the PD-1 group saw improved tumor growth inhibition efficacy. In another study, DCs and whole tumor cell lysates (TCL) were loaded in the gels, and there was a significant efficiency in inhibiting tumor growth in the Gel-DC-TCL + anti-PD-1 group.

Other cancer treatment strategies are looking at CD47 as a potential therapeutic target. CD47 is a transmembrane glycoprotein that emits a “do not eat” signal by binding to the signal regulatory protein α (SIRPα) on immune cells (Lian et al., 2019). Many cancers with poor prognosis express CD47 at high levels compared to normal cells (Chao et al., 2012; Lian et al., 2019). This signal is responsible for the escape of cancer cells from immune surveillance. Therefore, a self-assembling peptide LMY1 was created with a CD47 targeting motif, MMP-2 responsive peptide linker, self-assembly motif, and hydrophilic motif (Lv et al., 2022). The SAP first binds to CD47 on tumor cells followed by cleavage of the MMP-2 responsive peptide linker in the tumor immune microenvironment. This allows assembly into peptide-based nanofibers. The SAP nanofibers are then able to block subsequent interaction of CD47 and SIRPalpha, promoting the phagocytosis of tumor cells. In the subcutaneous Lewis lung carcinoma tumor model, combining LMY1 and anti-programmed death-ligand 1 (PD-L1) had the most significant antitumor efficacy and improved survival outcomes.

Sequestering immune checkpoint inhibitors in SAPs is a promising approach in cancer therapy. As illustrated by the previous example, combining multiple immunotherapeutic approaches is possible by using SAPs. A peptide nanofiber vaccine platform Coil29 was generated to include multiple epitopes to induce a coordinated antitumor immune response. Coil29 contains peptides with PEPvIII, B cell peptide epitope against EGFRvIII receptors, TRP2, a melanoma-associated CD8 T cell peptide antigen, and toxoid (Td) CD4 T cell epitope (Wu et al., 2022). After three subcutaneous immunizations with Coil29, the vaccine formulation containing three antigens PEPvIII, TRP2, and Toxoid (P/Tr/Td-fiber) and the Coil-29 with two antigens PEPvIII and Td (P/Td-fiber) both generated long-lasting PEPvIII-specific IgG responses compared to Complete Freund’s Adjuvant with the three antigens. The immunized mice were challenged with EGFRvIII-expressing B16vIII melanoma cells 2 weeks after final immunization. Tumor growth was significantly delayed in mice with P/Tr/Td-fibers and improved overall survival. When mice with B16vIII tumors were coadministered with P/Tr/Td-fibers and anti-PD-L1 and anti-CD47 there was significant tumor inhibition and long-term survival compared to the blockade group and the P/Tr/Td-fiber alone. Delivering multiple epitopes and immune checkpoint blockades in SAPs can aid in developing robust immune responses.

### 4.2 Cytokines and growth factors

Therapeutic strategies have also gone toward cytokine and growth factor interactions. Cytokines mediate the signaling among immune cells. There are different classes of cytokines, such as interleukins, interferons, and chemokines (Ramesh et al., 2013; Negishi et al., 2018). Some of the first approved cytokines were interferon α (IFN-α) as an adjuvant treatment for resected high-risk melanoma patients and high-dose IL-2 for metastatic renal cell cancer and melanoma. In cancer therapy, tumors can change their microenvironment by manipulating cellular and non-cellular components through complex signaling networks utilizing cytokines, chemokines, and growth factors to allow them to grow and spread (Baghban et al., 2020). In HNSCCs, the tumor immune microenvironment has been shown to impair tumor-infiltrating lymphocytes’ function and have immune-suppressive phenotypes, including myeloid-derived suppressive cells and T-regs (Chen et al., 2020). Dysfunctional tumor-infiltrating lymphocytes have decreased cytolytic activity and impaired production of effector cytokines such as IL-2, IFN-γ, and TNF-α (Zhang et al., 2020). Pro-inflammatory cytokines such as IL-2, IL-12, and IL-15 can improve antigen priming, increase the number of effector immune cells in the tumor immune microenvironment and enhance cytolytic activity (Berraondo et al., 2019). IL-2 can maintain T-regs to control the immune response and stimulate conventional T cells to promote the immune response (Abbas et al., 2018; Choudhry et al., 2018; Grasshoff et al., 2021). Unlike IL-2, IL-15 does not stimulate suppressive immunocytes such as T-regs (Saeed and Revell, 2001; Waldmann et al., 2020). IL-12 is crucial for the recruitment and effector functions of CD8 T cells and natural killer cells (Leonard et al., 1997; Del Vecchio et al., 2007; Nguyen et al., 2020; Mirlekar and Pylayeva-Gupta, 2021). As for growth factors, they stimulate cell proliferation, migration, and differentiation (Ren et al., 2019). These growth factors have been useful in regenerative medicine applications. Together, cytokines and growth factors can restore the intercellular communication needed to provide therapeutic potential.

Significant challenges arise when cytokines and growth factors are delivered with bolus or continuous administration (Pires et al., 2021). The small molecular size of cytokines and growth factors makes it difficult to localize them and can result in systemic toxicity outweighing their therapeutic efficacy (Baluna and Vitetta, 1997; Ren et al., 2019). Given the disadvantages of conventional systemic administration for cytokine therapy, a delivery vehicle capable of achieving the controlled release of cytokines would maximize their therapeutic potential while limiting the toxic systemic side effects and short half-life of these molecules (Berraondo et al., 2019; Conlon et al., 2019; Propper and Balkwill, 2022). Therefore, sequestering cytokines and growth factors in self-assembling peptides can mediate the release of these small signaling molecules.

Self-assembling peptides can be used to sequester cytokines. For instance, K-(SL)_6_-K-G-WKNFQTI (SLaM) was used to sequester chemokine (C-C) ligand 2 (CCL2), also known as monocyte chemoattractant protein-1 (MCP-1) from the extracellular matrix to reduce chemotaxis of monocytes and macrophages (Kim et al., 2020). Monocyte migration was reduced in the presence of SLaM hydrogel in contact with CCL2, and through ELISA found the hydrogel sequestered the majority of the CCL2 molecules in the solution. Using SAPs to sequester external signaling molecules can work as an adjunctive material for transplants or tissue regeneration without administering systemic immunosuppressants. Several cytokines can be delivered using SAPs. To demonstrate, a self-assembling domain and cell-adhesive fibronectin-derived RGDS sequence, also known as RGD, K(SL)_2_(SLRG)(SL)_3_K(GRGDS)(SLac) was used to release MCP-1 and IL-4 to foster a proangiogenic environment for tissue engineering applications (Kumar et al., 2015). MCP-1 had most of its release during the first 48 h, while IL-4 had prolonged release over 16 days. From the *in vivo* studies, SLac + MCP-1+IL-4 promoted infiltration, recruited macrophages, and polarized them to an M2 phenotype within and around hydrogel. This temporal release is not limited to proangiogenic cytokines; they can also be applied to pro-inflammatory applications such as cancer (Berraondo et al., 2019; Nash et al., 2021). The controlled release in SAPs can be beneficial in enhancing the half-life of cytokines and ensuring a robust immune response.

Spatiotemporal release can be achieved with the co-delivery of cytokines and growth factors in ischemic acute kidney injury (AKI). Patients with AKI have reduced kidney function and are at risk for chronic kidney disease. Many pro-inflammatory markers have been observed in AKI, including cytokines such as TNF-α and IL-1β, which can cause cell damage (Gao et al., 2013). Growth factors such as hepatocyte growth factor (HGF) have been reported to stimulate renal epithelial cell proliferation and induce tubular formation (Mao and Zhang, 2016). Therefore, the co-delivery of TNF-α neutralizing antibody and HGF in KLD2R/heparin hydrogel can enhance renal protective potential and reduce chronic renal fibrosis (Liu et al., 2020). A faster release of anti-TNF-α was achieved due to protein diffusion through the KLD2R hydrogel, while the slower release of HGF was due to a combination of heparin-binding affinity and molecular diffusion. This dual-drug delivery platform achieved sequential release kinetics of anti-TNF-α and HGF while promoting tissue repair. SAPs can allow for controlled spatiotemporal cytokine and growth factor release to augment an immune response.

## 5 Future directions

Self-assembling peptides can inherently contribute to therapeutics by integrating functional moieties. By knowing the disease pathology pathways SAPs can be modified to incorporate small molecules and receptor domains to improve disease state. Although functional moieties can be incorporated into SAPs, it is difficult to precisely control and predict secondary and supramolecular structures (Scanlon, 2008; Pitz et al., 2022). Therefore, more advanced computational modeling must be developed to accurately predict the structure of self-assembling peptides (Chang et al., 2022; Pitz et al., 2022).

The ability to modify peptides in SAPs makes it possible for large-scale production. In dental applications, it is common to treat early caries with fluoride to repair tooth enamel. A commercially available P_11_-4, Curodont™ Repair, was combined with fluoride for the non-invasive treatment of early occlusal enamel lesions (ClinicalTrials.gov: NCT02724592). They found the combination of P_11_-4 with fluoride facilitated biomimetic mineralization and was a safe and effective treatment for early carious lesions (Alkilzy et al., 2018). Current studies are looking into SAPs as an adjunct during surgery. There is a risk for hemorrhage by using Transoral Robotic Surgery for human papillomavirus positive early-stage oropharyngeal squamous cell carcinoma (Leonhardt et al., 2012; Moore et al., 2012; Chen et al., 2015). A hemostatic agent from the RADA16 family, PuraBond^®^, was used as an adjunct during Transoral Robotic Surgery (Gupta et al., 2022). Preliminary findings showed none of the patients developed primary or secondary hemorrhage post-transoral robotic surgery in conjunction with PuraBond^®^. These findings were based on a small cohort; further large-scale studies are needed to determine whether patients receive clinical benefit from the hemostatic and regenerative properties of PuraBond^®^ (ClinicalTrials.gov: NCT05405907).

Immunomodulators such as cytokines traditionally have rapid clearance. The usual systemic delivery of ICIs have been associated with severe immune-related adverse events and even death; therefore, sequestering these modulators in SAPs can control drug release (Newton et al., 2019). Although maintaining modulators is important to sequester, it is imperative to consider the rate of degradation of the biomaterial. If a significant amount of cytokines are released at once, it can result in toxicity and, depending if there is a robust immune response, a cytokine storm (Baldo, 2014; Pires et al., 2021). Future work should consider having the temporal release of modulators that can strategically activate immune cells at certain times, which will be necessary with combination therapy.

There are commercially available SAPs being used as scaffolds and delivery vehicles. RADA16 has been commercialized as PuraMatrix™^,^ which forms 3D hydrogel-forming nanofibrous structures to support the attachment of various cell types, tumor cell migration and invasion, and *in vivo* analysis of tissue regeneration. PuraMatrix™, in combination with mesenchymal stromal cells, was used as an epicardial coating of the heart and noted improved global cardiac function and decreased ventricular dilatation (Ichihara et al., 2018). Using these readily available SAPs allows for further modification in several other applications. To date, there are no clinical trials or FDA-approved self-assembling peptides for immunotherapy applications, although clinical trials of these materials for tissue regeneration are ongoing (ClinicalTrials.gov: NCT05127889, NCT05206539). Given the promise SAPs have shown for immunomodulation in the preclinical setting, we anticipate clinical applications of these materials in the future.

## 6 Conclusion

Self-assembling peptides are versatile materials that allow for tailoring peptide sequences to house antibodies, cytokines, and small molecules for applications in tissue engineering, immunotherapy, and drug delivery. SAPs can mimic the natural framework and are tunable to include biologically active systems. The adaptable nature of these peptides allows for the modification of their chemical composition to include modulators. A critical part of SAPs is maintaining biomaterial integrity to build the foundation of tissue growth or release immunomodulators for drug delivery and immunotherapy applications. To develop a precise SAP structure and targeting ability, advanced computational modeling is needed. The next-generation of SAPs should consider integration of spatiotemporal motifs for combination treatment.
